# A systematic review of provider-and system-level factors influencing the delivery of cardiac rehabilitation for heart failure

**DOI:** 10.1186/s12913-021-07174-w

**Published:** 2021-11-24

**Authors:** Paulina Daw, Thomas M. Withers, Jet J. C. S. Veldhuijzen van Zanten, Alexander Harrison, Colin J. Greaves

**Affiliations:** 1 School of Sport, Exercise & Rehabilitation Sciences, University of Birmingham, Edgbaston, Birmingham, B15 2TT UK; 2 Health Sciences, University of York, York, UK

**Keywords:** Cardiac rehabilitation, Heart failure, Implementation science, Systematic review

## Abstract

**Background:**

There is a longstanding research-to-practice gap in the delivery of cardiac rehabilitation for patients with heart failure. Despite adequate evidence confirming that comprehensive cardiac rehabilitation can improve quality of life and decrease morbidity and mortality in heart failure patients, only a fraction of eligible patients receives it. Many studies and reviews have identified patient-level barriers that might contribute to this disparity, yet little is known about provider- and system-level influences.

**Methods:**

A systematic review using narrative synthesis. The aims of the systematic review were to a) determine provider- and system-level barriers and enablers that affect the delivery of cardiac rehabilitation for heart failure and b) juxtapose identified barriers with possible solutions reported in the literature. A comprehensive search strategy was applied to the MEDLINE, Embase, PsycINFO, CINAHL Plus, EThoS and ProQuest databases. Articles were included if they were empirical, peer-reviewed, conducted in any setting, using any study design and describing factors influencing the delivery of cardiac rehabilitation for heart failure patients. Data were synthesised using inductive thematic analysis and a triangulation protocol to identify convergence/contradiction between different data sources.

**Results:**

Seven eligible studies were identified. Thematic analysis identified nine overarching categories of barriers and enablers which were classified into 24 and 26 themes respectively. The most prevalent categories were ‘the organisation of healthcare system’, ‘the organisation of cardiac rehabilitation programmes’, ‘healthcare professional’ factors and ‘guidelines’. The most frequent themes included ‘lack of resources: time, staff, facilities and equipment’ and ‘professional’s knowledge, awareness and attitude’.

**Conclusions:**

Our systematic review identified a wide range of provider- and system-level barriers impacting the delivery of cardiac rehabilitation for heart failure, along with a range of potential solutions. This information may be useful for healthcare professionals to deliver, plan or commission cardiac rehabilitation services, as well as future research.

**Supplementary Information:**

The online version contains supplementary material available at 10.1186/s12913-021-07174-w.

## Background

Heart failure is a debilitating progressive clinical syndrome, that due to increasing life expectancy and more widespread adoption of a western lifestyle has seen a steady increase in prevalence across the globe [1]. The cost of treating patients with heart failure by the National Health Service is estimated at two billion pounds per year, with most of the cost associated with hospital admissions [2]. There is also a substantial human cost of heart failure, as many patients experience a diminished quality of life related to their illness [3]. Improving health-related quality of life is a fundamental aim of heart failure management [4].

Key strategies for improving health-related quality of life include self-management of symptoms and psychological consequences of heart failure and exercise-based rehabilitation of physical functioning, all of which are part of comprehensive cardiac rehabilitation programmes. Several trials and systematic reviews have confirmed the safety and effectiveness (reduction in hospital admissions and improvement in health-related quality of life) of cardiac rehabilitation for heart failure [5–7]. Thus, cardiac rehabilitation programmes are an effective and cost-effective strategy for improving health-related quality of life in people with heart failure [8, 9].

Despite the strong evidence for effectiveness, according to a recent global survey, cardiac rehabilitation is available in only half the countries of the world [10]. Furthermore, even in countries that do offer cardiac rehabilitation services, coverage is low. Globally only 30% of eligible patients access cardiac rehabilitation [11] and there are large regional variations in the content of cardiac rehabilitation programmes [12]. The European Cardiac Rehabilitation Inventory Survey 2010 [13] also highlighted that less than 20% of patients with heart failure receive cardiac rehabilitation.

The low proportion of eligible patients receiving cardiac rehabilitation may reflect a lack of service availability or it may reflect low uptake by patients of services. For example in the UK, uptake of cardiac rehabilitation is estimated to be around 50% on average, with lower uptake in women, ethnic minorities and people living in rural areas and areas of high deprivation [14]. There is a large body of evidence exploring patient-level factors impacting cardiac rehabilitation enrolment/attendance, compliance/adherence, completion and drop-out rates amongst general cardiac population [15–23]. These factors include distance required to travel, financial constraints and work obligations [24]. However, to the best of our knowledge, there have been no systematic reviews of non-patient level factors – i.e., provider- and system-level barriers affecting the delivery of cardiac rehabilitation for patients with heart failure.

The current systematic review, therefore, aimed to answer the following research question: ‘What are the factors influencing the offer, referral, delivery, implementation, and provision of cardiac rehabilitation for heart failure?’. The purpose of the study was to identify and qualitatively describe barriers and enablers affecting the delivery of cardiac rehabilitation for patients with heart failure.

## Methods

The systematic review has been registered with PROSPERO (CRD42019153247), conducted according to Guidance on the Conduct of Narrative Synthesis in Systematic Reviews [25] and reported in concordance with PRISMA guidance [26].

### Inclusion and exclusion criteria

The scope for the systematic review is illustrated in Table 1.

**Table 1:** PICOS search strategy

PICOS	Definition
Population	Services and professionals working with patients with heart failure
Intervention	‘A coordinated and structured programme designed to remove or reduce the underlying causes of cardiovascular disease’ to ‘include a range of interventions with health education, lifestyle advice, stress management and physical exercise components’ [27–30].
Comparison	None
Outcome	Barriers and enablers
Study type	Any empirical

### Search strategies

The full search strategy is provided in Additional file 1. The following databases were searched using a combination of free-text search terms and controlled vocabulary (Medical Subject Headings): MEDLINE (OVID interface), Embase (OVID interface), PsycINFO (OVID interface), CINAHL Plus, and EThoS and ProQuest libraries. The only exclusion criterion applied to the search strategies was for studies in languages other than the English language.

### Study selection

PD conducted all searches and the initial screening of all titles and abstracts; CG and TW screened 20% each of the total titles and abstracts. Following the initial screening, PD read the full text of all potentially eligible articles. CG and TW reviewed 50% each of the total of full-length articles against the eligibility criteria. To ensure saturation in sources, extensive backward and forward citation tracking was applied to reference lists of relevant articles and key texts. Any discrepancies in selection were discussed between the reviewers and a fourth reviewer (JVvZ) was available for arbitration if needed. No additional information had to be sought from study authors to inform eligibility decisions. The review authors were not blind to the journal titles, study authors or institutions of the full-text articles.

### Study appraisal

We used four different quality assessment tools for different study designs in line with the National Institute for Health and Care Excellence manual on developing guidelines [31]. The chosen study appraisal tools are listed in Table 2. Using the most suitable quality assessment tool, a total numerical score obtained for each study was re-calculated into percentages and assigned into the following categories of quality: low (below 20%), low-to-medium (20–44%), medium (45–69%), medium-to-high (70–89%) and high (above 90%). The quality assessment was conducted by PD and TW who independently scored all of the included studies.

**Table 2:** Characteristics of included studies and quality assessment tools/scores

Study details					Population	Quality assessment
*Author (year)*	*Study design/methods of data collection*	*Country/setting*	*Sample size*	*Study/report aim*	*Healthcare professional/s*	*Tool (score)*
Achttien et al. (2015) [32]	Guideline review Document analysis (Dutch and European CR guidelines and position statements), systematic review and expert panel	CR centres in Netherlands offering exercise-based CR	N/R	To develop evidence-based clinical algorithms that can serve as best practice standards for prescription and evaluation of exercise-based CR in patients with coronary artery disease and chronic HF	Multidisciplinary expert panel (cardiologists, physiotherapists, sports physicians, occupational physicians, rehabilitation physician, human movement scientist and health informatician)	AACODS checklist [33] (medium-to-high)
Dalal et al. (2012) [34]	Cross-sectional survey Two-stage, postal questionnaire-based national survey (the stage 1 questionnaire responses were 224 out of 277 and 17 out of 24 for stage 2)	CR programmes in England, Wales and Northern Ireland	*n* = 224 at stage 1 and *n* = 17 at stage 2	To determine why so few patients with chronic HF in England, Wales and Northern Ireland take part in CR	Service managers and other heartcare professionals responsible for the CR service/team	Centre for Evidence-Based Management survey questionnaire study checklist [35] (medium)
Frolich et al. (2010) [36]	Observational, non-comparative case study Surveys, before and after patient performance measurements, semi-structured interviews and observations (with key informants, including the leadership of the hospital and healthcare centres, a leading representative for the GPs, the project leaders, health professionals in the hospital and in the healthcare centre, and GPs)	Quality improvement project set up in Denmark: Bispbjerg University Hospital, the City of Copenhagen and the GPs in Copenhagen	*n* = 44 GPs answered the mailed questionnaire	To describe the process and results of a project that led to the development of new management practices and improvement of existing ones to support integrated care between three healthcare organisations	Two specialists (in geriatrics and internal medicine), specialist physiotherapist, nurse specialist, project leaders, hospital management, department leadership, leadership of the healthcare centre, representatives of the GPs, ‘a steering committee’ and four working groups	National Heart, Lung, and Blood Institute Quality Assessment Tool for Before-After (Pre-Post) Studies With No Control Group [37] (medium)
Golwala et al. (2015) [38]	Observational, prospective Get With The Guidelines–heart failure (GWTG-HF) registry and quality improvement programme Used the GWTG-HF database to determine the contemporary proportional use, temporal trends, and major factors associated with referral for CR at discharge among eligible patients with HF	Various institutions representing community hospitals and tertiary-care referral centres from all USA geographic regions	*n* = 338	To assess proportional use, temporal trends, and factors associated with CR referral at discharge among patients admitted with decompensated HF	Hospital staff ordinarily looking after HF patients	National Heart, Lung, and Blood Institute Quality Assessment Tool for Before-After (Pre-Post) Studies With No Control Group [37] (medium)
Nguyen et al. (2013) [39]	Observational, retrospective cohort study Database analysis (multivariate logistic regression to examine patient characteristics, in-hospital diagnosis, clinical events, investigations associated with CR referral)	Hospitals in Canada, Ontario	*n* = 11	To assess CR referral rates during index hospitalization (report the frequency and temporal trends of CR referral rates in Ontario, describe the factors associated with CR referral and examine the use of evidence-based medical therapies and their relationship with CR referral before hospital discharge)	Hospital staff from 11 Canadian sites reporting to the Global Registry of Acute Coronary Events (GRACE) database	Critical Appraisal Skills Programme Cohort Study Checklist [40] (medium-to-high)
Palmer et al. (2020) [41]	National online cross-sectional survey (365 registered programmes were contacted and 165 healthcare professionals completed the survey)	Cardiac rehabilitation programmes in Australia taking place in community settings and accepting HF patients Programmes were excluded if their rehabilitation programme was conducted within an inpatient hospital setting	*n* = 165 healthcare professionals completed the survey	The primary aim of the study was to identify clinician perceived barriers to engagement in rehabilitation for people with HF	Participants were clinicians such as registered nurses or physiotherapists working as the programme coordinators	Centre for Evidence-Based Management survey questionnaire study checklist (medium)
Piepoli et al. (2019) [42]	Survey questionnaire study Sub-analysis of the web-based Exercise Training in HF (ExtraHF) survey	Cardiac centres from the European Society of Cardiology affiliated countries	*n* = 172	To investigate the regional variations in the implementation and prioritisation of exercise training programmes; to identify specific/local barriers to implementation	Cardiologists, nurses, psychologists, exercise physiologists/therapists, dieticians, physiotherapists	Centre for Evidence-Based Management survey questionnaire study checklist [35] (medium)

*CR* Cardiac rehabilitation, *HF* Heart failure, *N/R* Not reported

### Data extraction

PD extracted study characteristics and any relevant data on factors influencing delivery of cardiac rehabilitation from the included studies using a data extraction template. Data extraction for all included studies was verified by CG and TW. The extracted study characteristics included: author, year, study design/methods of data collection, country/setting, sample size, study/report aim and healthcare professional population. Extraction of the data pertaining to provider- and system-level barriers and enablers associated with the delivery of cardiac rehabilitation for heart failure included first-order constructs (data from the original study participants) and second-order constructs (assumptions and observations made by the studies’ researchers). The review team only included reported data (i.e., a lack of barrier was not entered as an enabler unless the article clearly stated that). Passages of text describing barriers and enablers were inputted and organised in the nVivo software [43] and summarised into a table available in the Additional file 2.

### Data synthesis

In developing our analytic approach, we followed guidance on the selection of qualitative evidence synthesis methods for health technology assessments of complex interventions [44] and the seven-domain RETREAT framework [45]. The following components of the framework were considered: the type of the review question, the review’s purpose and the targeted audience, the timeframe, availability of resources and expertise, and the type of available data. Consequently, we conducted a narrative review of the qualitative data, using the following tools and techniques as described by Popay et al. For building preliminary syntheses – textual descriptions, tabulation, groupings and clusterings and thematic analysis, for exploring relationships within and between studies – concept triangulation and consideration for variability in outcomes, study design and study population, and for assessing robustness of findings – critical reflection. Additionally, categories identified during the thematic analysis were further considered according to the level of influence from the social ecological model [46].

### Thematic analysis

All data relevant to the research question was entered into the nVivio software. The verbatim text of first and second-order constructs representing barriers and enablers was organised thematically using thematic coding procedures described by Braun and Clarke [47]. First and second-order constructs were given the same weight in the final analysis. The coding scheme emerged inductively following reading and rereading of the original data sources and discussions between the core review team (PD and CG). The final coding scheme consisted of a small number of overarching categories and a larger set of more granular themes within each category. The identified themes were further analysed in terms of their frequency and prominence (identifying the most common themes across the data set and their spread).

### Triangulation protocol

A triangulation protocol was used to summarize similarities and differences between different data sources [48]. Each theme was considered in each data source and categorised as being in agreement, partial agreement or dissonance. An additional category (isolation) was created for themes that were neither confirmatory nor contradictory, as they simply added a concept that was not identified in other studies. In case of disagreement between data sources, further data within the articles (e.g. year of publication, differences in populations or methods used) was considered as potential explanations of such discrepancies.

## Results

**Fig. 1:**
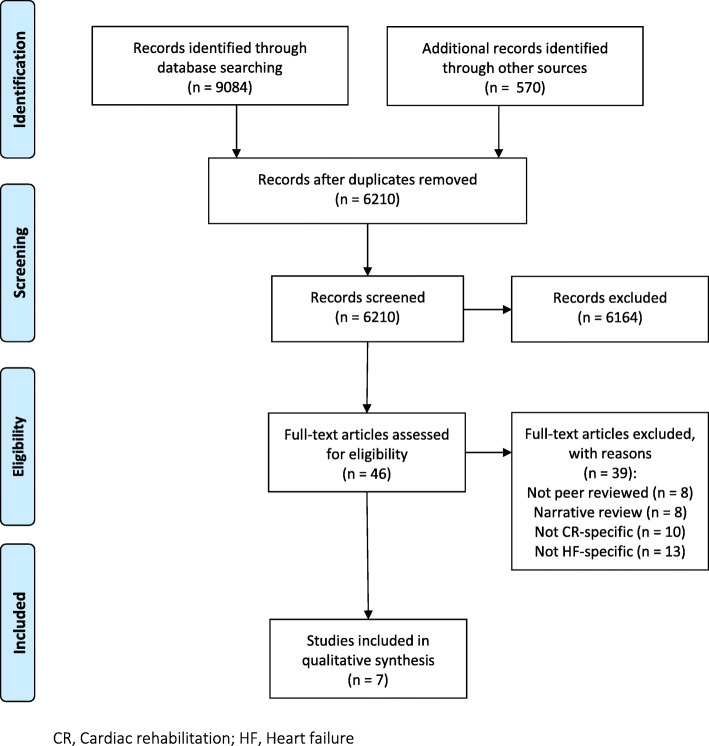
Flow diagram by PRISMA of included studies

All searches were conducted in October 2019 by PD and updated in March 2021. The searches identified 9654 articles, of which 3444 were duplicates. Following the screening of titles and abstracts of 6210 articles, 46 full-text articles were obtained, and seven articles were included for analysis [32, 34, 36, 38, 39, 41, 42]. The full search results are presented in Fig. 1.

### Study characteristics

The characteristics of the included studies and the quality assessment tools/scores are presented in Table 2. There was a little demarcation between studies in terms of setting (centre-based cardiac rehabilitation programmes taking place in hospitals or community settings), healthcare professionals involved in the study (members of multidisciplinary teams that ordinarily care for patients with heart failure), methods of data collection (mostly qualitative methods utilising document analysis, survey questionnaires, interviews, focus groups and observations) or evidence quality (medium to medium-to-high). The included studies were published between 2010 and 2020 and represented mostly European healthcare systems (i.e., Denmark, Netherlands, the UK and the European Society of Cardiology affiliated countries) or western healthcare systems (i.e., Australia, Canada and the USA). Five studies were rated as being of medium quality and two were rated as medium-to-high quality.

### Thematic analysis

During the process of thematic analysis, the identified barriers were organised into nine categories and 24 themes. The same categories, except one (‘the origins of cardiac rehabilitation and previous practices’) emerged in the thematic analysis of reported enablers; the enablers were further divided into 26 themes. Table 3 contains a summary of the thematic analysis, the main analysis used to analyse available data. This table lists the identified categories and themes, highlights each theme frequency and coverage and, where possible, matches a theme related to a barrier with a counteracting enabler.

**Table 3:** Barriers to and enablers of delivering cardiac rehabilitation to patients with heart failure identified in our thematic analysis

Overarching categories	Barriers/factors preventing delivery of cardiac rehabilitation (theme frequency/coverage)	Enablers/factors promoting delivery of cardiac rehabilitation (theme frequency/coverage)
The origins of CR and previous practices	The outdated practise of bed rest [39, 42]	
Evidence-base	Poor evidence-base supporting CR for HF [34, 38]	Sufficient evidence-base supporting CR for HF [38, 39]
Guidelines	Guidelines not tailored to the end-user [32, 34]	Better tailoring of guidelines [32, 34]
	Volume and complexity of guidelines [32, 42]	Translating guidelines into clinical algorithms [32]
	Lack of inclusion of CR in local guidelines [42]	Guideline endorsement [38, 39]
		Cross-institutional guidelines [36]
		Guideline implementation [42]
Education	Lack of formal education on exercise training [42]	Education programmes on the importance of exercise training [42]
		Knowledge sharing opportunities [36, 38]
		Awareness-raising [39, 41]
Medical insurance	Lack of medical insurance cover [38]	Medical insurance eligibility criteria and sufficient cover [38]
Resources	Lack of resources: time, staff, facilities and equipment [32, 34, 41, 42]	Adequate resources: time, staff, facilities and equipment [42]
The organisation of healthcare system	Lack of commissioning [34, 42]	Sufficient commissioning [38, 42]
	Blurred professional roles [34, 42]	Clear professional roles and responsibilities [38, 42]
	Lack of integration between organisations [36, 42]	Better integration between organisations [36, 42]
	Lack of patient pathways [34, 41, 42]	Referral system [39]
	Inadequate IT systems [32]	Adequate IT systems [32, 42]
	Lack of integration between departments [36]	Better integration between departments [36, 42]
	Lack of care standardisation [42]	Care standardisation [36]
	Lack of implementation strategies [38]	
	Lack of referrals [34, 41]	
		Healthcare legislation [38]
		Performance and target measures [39]
		Use of clinical algorithms [32]
The organisation of CR programmes	Lack of different modes of delivery [34, 41]	Availability of different modes of delivery [34, 38, 41]
	Lack of programmes [42]	Availability of programmes (specialised and community-based) [42]
	Limiting eligibility criteria [38]	Broadened eligibility [42]
	Difficult to choose a suitable programme [36]	
	Confusing referral procedures [36]	
Healthcare professional	Poor professional’s knowledge, awareness and attitude [32, 38, 39, 41]	Sufficient professional’s knowledge, awareness and attitude [36, 38, 39, 41]
	Safety concerns [38, 39, 41]	
		Improving the doctor-patient relationship [42]

*CR* Cardiac rehabilitation, *HF* Heart failure

**Fig. 2:**
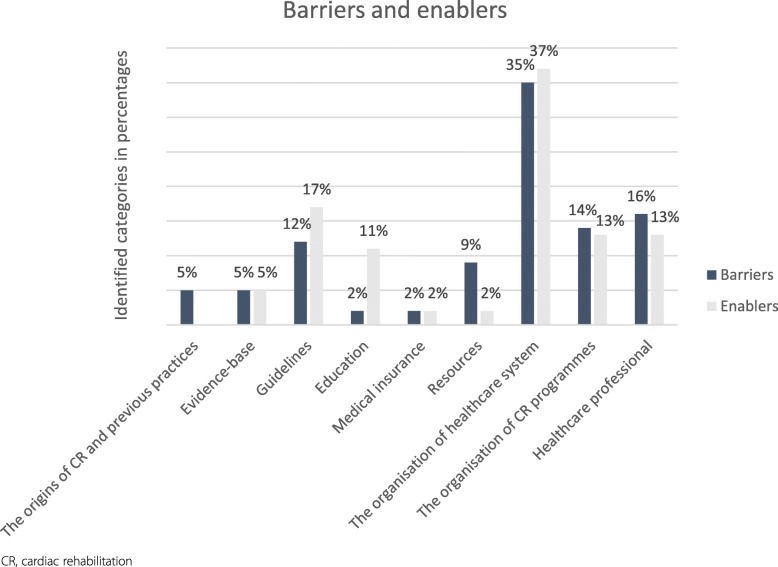
Identified categories in percentages

‘The organisation of healthcare system’ was the most frequent category for both barriers (15 instances) and enablers (15 instances) and this category was mentioned at least once in all of the included articles. The other most frequent categories related to barriers were ‘the organisation of cardiac rehabilitation programmes’, ‘healthcare professional’ and ‘guidelines’. The same categories were the most frequent categories describing enablers. Themes pertaining to barriers that were quoted most frequently in the included studies were ‘lack of resources: time, staff, facilities and equipment’ and ‘professional’s knowledge, awareness and attitude’. The latter was also the most frequently identified enabler. Figure 2 apportions the identified categories relating to barriers and enablers.

### Triangulation of themes across the data sources

Convergence analysis (Table 4) revealed that 50% (12) of themes related to barriers and 53% (14) of themes related to enablers appeared as isolated concepts. There was agreement or partial agreement for 50% (12) of the identified barriers and dissonance was identified for 8% (2): ‘poor professional’s knowledge, awareness and attitude’ and ‘safety concerns’ – themes that showed the most complex convergence relationship (agreement, partial agreement and dissonance).

**Table 4:**
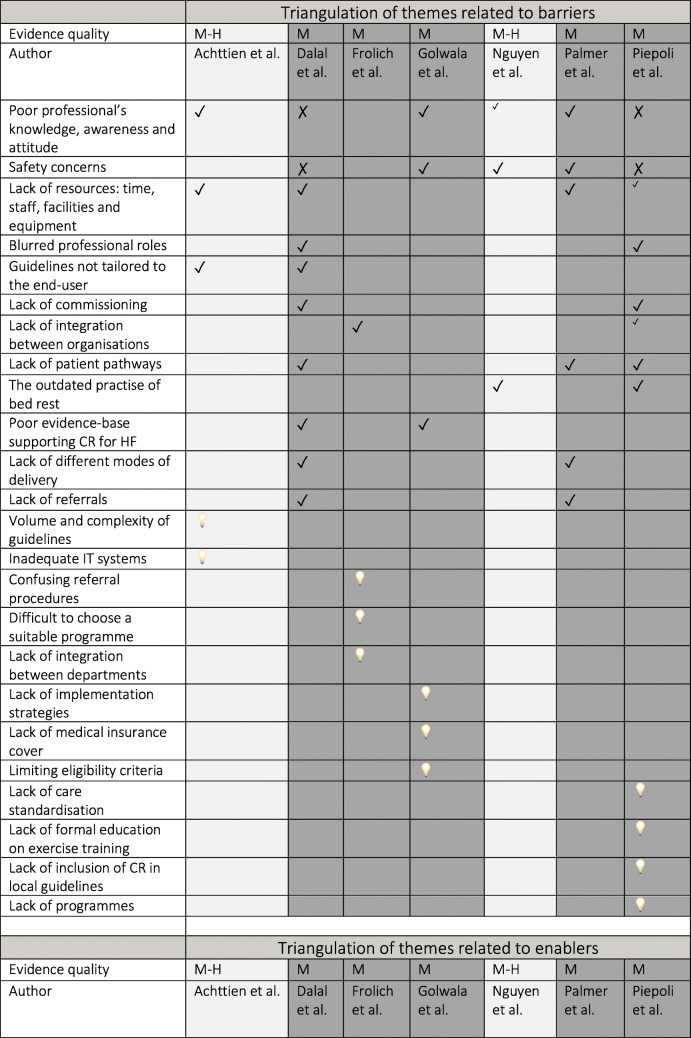

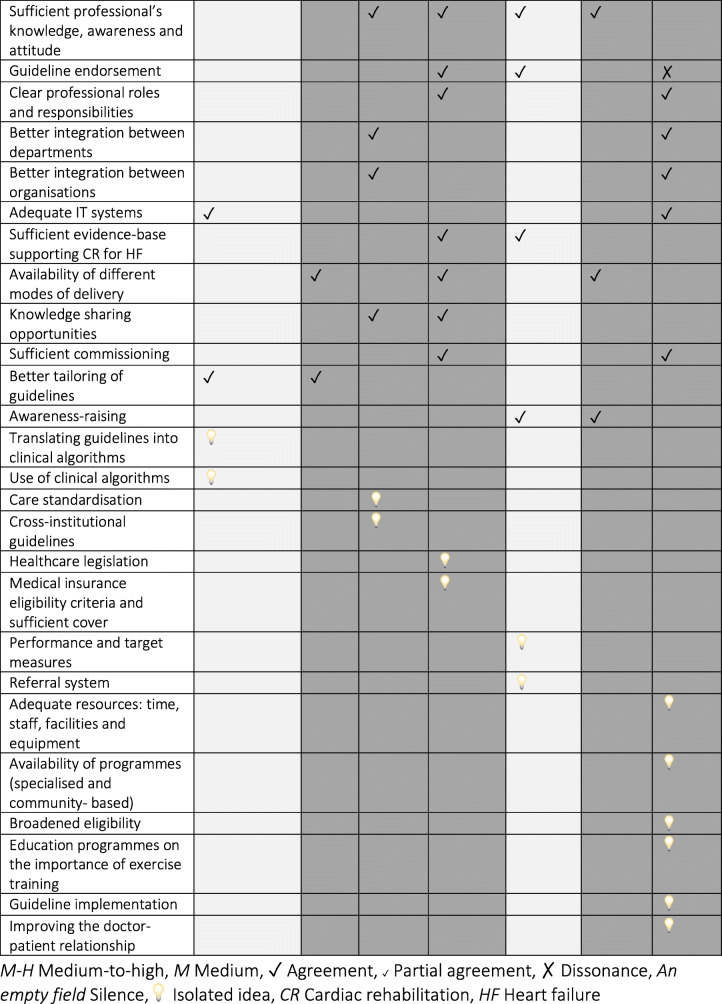
Triangulation of reported barriers and enablers across the data sources

*M-H* Medium-to-high, *M* Medium, ✓ Agreement, ^✓^ Partial agreement, ✗ Dissonance *An empty field* Silence, □ Isolated idea, *CR* Cardiac rehabilitation, *HF* Heart failure

Piepoli et al. concluded that ‘perceived lack of importance, safety concerns, physicians not being confident or not having sufficient skill or knowledge and uncertainties about the usefulness all played a marginal role’ [42]. Similarly, Dalal et al. found that ‘more than half (54%) of the centres expressed confidence in the skill mix and knowledge of their staff to provide cardiac rehabilitation in heart failure’, as well as that ‘a lack of evidence on safety or clinical benefit was not a factor that influenced most centres’ ability to offer cardiac rehabilitation’ [34].

Thus, Piepoli et at. and Dalal et al. were in agreement about a marginal influence of ‘poor professional’s knowledge, awareness and attitude’ and ‘safety concerns’, that was at odds with the remaining data sources, which recognised those as substantial barriers. Additionally, Piepoli et al. concluded that ‘lack of resources: time, staff, facilities and equipment’ was a barrier affecting non-Western regions of the European Society of Cardiology affiliated countries only. This partial agreement with two other studies might be linked with Piepoli et al. considering in their analysis several distinct geographical areas and therefore capturing a more nuanced picture in the results.

Fifty-seven percent (4) of sources were aligned regarding the top potential factor positively impacting the delivery of cardiac rehabilitation for heart failure (i.e., ‘professional’s knowledge, awareness and attitude’). Twelve (46%) enabler themes were classified as being in agreement with at least one additional data source. Only one (4%) dissonant relationship was identified amongst themes related to enablers and this theme was linked to ‘guideline endorsement’. Piepoli et al. highlighted that barriers to the delivery of cardiac rehabilitation ‘cannot be overcome by the development of different guidelines for the different geographical areas (Southern/Northern/Western/Eastern/extra-EUR), but by a better implementation of the existing ones’ [42]. This was in conflict with two other studies, which reported guideline endorsement as a potentially enabling factor.

## Discussion

The systematic review identified a wide range of provider- and system-level barriers and enablers affecting the delivery of cardiac rehabilitation for heart failure and linked the identified barriers with possible solutions. The broad array of factors identified may reflect the complexity of the phenomenon or it may reflect the range of healthcare systems and implementation contexts studied. Encouragingly, most of the identified barriers were matched with potential ‘enablers’ or solutions.

The most prevalent barriers were ‘poor professional’s knowledge, awareness and attitude’, ‘lack of resources: time, staff, facilities and equipment’ and ‘safety concerns’. Interestingly, the most prevalent themes also showed some dissonance, with one of the most recent studies [42] presenting a more nuanced and updated picture relating to those factors. Namely, that lack of resources might not be as much of a barrier in Western regions of the European Society of Cardiology affiliated countries as opposed to more poorly-resourced areas and that professionals’ knowledge and safety concerns may no longer be as prevalent as they have been previously reported [49]. The latter dissonance might be linked with changing attitudes of healthcare professionals as a result of a gradually improving evidence-base for offering cardiac rehabilitation to heart failure patients [50].

The majority of identified barriers were consistent with literature outlining more generic barriers to implementation of healthcare services. Examples of this are the system, staff and intervention-level barriers affecting implementation of novel interventions identified by Geerligs et al. [51] or barriers to change identified by the National Institute for Health and Care Excellence (e.g. staff awareness, knowledge, workforce skills, resources and political environments) [52]. A barrier identified in the review that might be particularly pertinent to the delivery of cardiac rehabilitation for heart failure patients that has not been considered extensively in other literature is ‘the origins of cardiac rehabilitation’. The awareness of healthcare staff of the benefits of cardiac rehabilitation (as opposed to the outdated practice of bed rest) is a strong predictor of cardiac rehabilitation referral [53].

The identified categories of barriers and potential solutions fit well with the social ecological model which has previously been used to identify influences impacting healthcare delivery at several different levels (Table 5). These include the macrosystem encompassing widely shared cultural/social values, beliefs, customs and laws (e.g. public policies, enabling environments), the exosystem capturing the indirect environment (e.g. economic system, political system, educational system, governmental system, community-level influences), the microsystem describing the interpersonal environment (e.g. a small group of professionals who work together on a regular basis) and the mesosystem capturing the interactions between microsystem and exosystem (e.g. organisation-level influences). The most granular level of influence is the individual level, in this case, understood as an intrapersonal environment (e.g. a healthcare professional providing care to individual patients).

**Table 5:** Social ecological model

Level of influence	Barriers	Potential solutions
Individual	Healthcare professional	• Establishing inter-professional collaboration forums (e.g. working groups, knowledge-sharing meetings)
		• Developing collaborative relationships between health professionals looking after HF patients
Microsystem	The organisation of CR programmes	• Using new delivery systems such as telemedicine
		• Providing choice between hospital-based group rehabilitation and home-based individual programmes
		• Providing feedback to programmes regarding the management of their HF patients
Mesosystem	The organisation of healthcare system	• Providing integrated healthcare
		• Developing local patient pathways
		• Using automatic referral systems
Exosystem	Education	• Education programmes for healthcare professionals on the importance of exercise training
	Medical insurance	• Better collaboration with healthcare authorities
		• Increasing insurance coverage
	Resources	• Inclusion of CR for HF in local commissioning contracts
		• Changes to healthcare systems that improve access to CR by removing some of the financial constraints (such as accountable care organisations under the new Affordable Care Act in the United States)
Macrosystem	The origins of CR and previous practices	• Initiatives influencing awareness of the importance of CR (e.g. the Cardiac Rehabilitation Network of Ontario)
	Evidence-base	• Increasing the evidence-base confirming the benefits and safety of CR in patients with HF (especially HFpEF)
	Guidelines	• Development of cross-institutional guidelines
		• Combining and translating guidelines into clinical algorithms (to reduce practice variation and increase guideline adherence)
		• Better implementation of existing guidelines

*CR* Cardiac rehabilitation, *HF* heart failure, *HFpEF* Heart failure with preserved ejection fraction

Barriers to the delivery of cardiac rehabilitation for heart failure patients are varied and multi-levelled and overcoming them will involve changes at different levels. This reflects the suggested ‘re-engineering of health care system’ and ‘progressive policy’ in the recently published Journal of the American College of Cardiology expert panel report [54]. Individual and microsystem-level initiatives include creating inter-professional knowledge-sharing opportunities or in-house monitoring and evaluation of the management of heart failure patients. These solutions can be implemented by individual cardiac rehabilitation teams.

An example of a practical solution from the mesosystem of influence was introducing an automated referral system to mitigate barriers linked with poor clinical knowledge. Such organisational level solutions may also facilitate the development of local patient pathways (which in turn may lead to the provision of more integrated healthcare).

Exosystem and macrosystem-level solutions related to the availability of resources and the creation of further evidence require collaborations between many different stakeholders and rely on policy-level changes and improvements (e.g., development of cross-institutional guidelines or increasing insurance cover).

In recent years, healthcare systems have been described as complex and adaptive [55]. A change in one part of the system can lead to changes to other components, for example offering education to healthcare professionals on the benefits of cardiac rehabilitation in heart failure patients may lead to development of inter-professional collaborations or inspire service providers to use novel delivery systems.

### Strengths and limitations

To the best of our knowledge, this is the first systematic review investigating provider- and system-level factors affecting the delivery of cardiac rehabilitation for heart failure. The review applied robust methods, i.e., systematic search strategy, second coding of study selection and study quality procedures, use of comprehensive narrative synthesis techniques that included thematic analysis and triangulation of identified themes to maximise depth and robustness of the findings. Additionally, the included studies used different methodologies leading to triangulation of available data and increasing rigour of the systematic review findings.

Despite applying a very inclusive search strategy the review identified only seven studies meeting the inclusion criteria. The paucity of empirical studies and/or relatively poor quality of empirical data limits the findings and increases the possibility of a publication bias being present in the final synthesis. Additionally, although including second-order constructs increased the overall amount of data, the origins and robustness of the second-order constructs were difficult to establish.

Due to limitations of the data reported in the reviewed literature, we were unable to consider how representative the sample was of professionals involved in the delivery of cardiac rehabilitation for heart failure. However, we were able to identify that the sample was restricted mainly to European and Western healthcare systems. Therefore the generalisability of the identified barriers and enablers is limited to this context. Furthermore, the literature that we reviewed did not report characteristics of the patient populations served or consider how barriers might vary depending on patient characteristics (e.g. some healthcare professionals may be less willing to invite more frail patients for cardiac rehabilitation).

### Future research

Further research is needed to identify barriers in other healthcare systems and in a wider, more clearly defined range of healthcare professionals. Future implementation studies could also seek to identify any barriers and enablers that apply differently to different patient groups. Further research is also needed to qualitatively investigate barriers that are unique to the heart failure population (e.g. the origins of cardiac rehabilitation) and barriers that showed divergent relationships between sources included in our review (e.g. the impact of professional’s knowledge, guidelines, safety concerns and lack of resources).

The gaps in the literature, uncovered by the systematic review, confirmed a continuing dearth of implementation studies on the topic of cardiac rehabilitation for heart failure and an ongoing need for further high quality research that goes beyond patient-level factors affecting the delivery of cardiac rehabilitation for heart failure. Such research is acutely needed in the light of initiatives to improve access to and uptake of cardiac rehabilitation for heart failure, such as the National Health Service Long Term Plan that aims to increase the proportion of eligible heart failure patients accessing cardiac rehabilitation from less than 10 to 33% by 2028 [56, 57].

## Conclusions

This systematic review identified a broad range of provider- and service-level factors affecting the delivery of cardiac rehabilitation for heart failure. The identified barriers and enablers operate on multiple levels of influence from the knowledge and views of individual healthcare professionals to the organisation of cardiac rehabilitation teams and the wider healthcare system. Consequently, efforts to increase the delivery of cardiac rehabilitation for patients with heart failure will likely require intervention at all these levels. Strategies for improving delivery of cardiac rehabilitation for heart failure may include increasing inter-professional collaboration, providing choice between hospital and home-based rehabilitation programmes, inclusion of cardiac rehabilitation for heart failure in local commissioning contracts and staff-education initiatives to raise awareness of the importance of cardiac rehabilitation and of the evidence-base on the benefits and safety of cardiac rehabilitation in patients with heart failure.

## Supplementary Information

**Additional file 1.** MEDLINE Ovid search strategy.

**Additional file 2.** Provider- and system-level barriers and enablers identified in the literature.

## Data Availability

The datasets used and/or analysed during the current study are available from the corresponding author on reasonable request.

## Abbreviations

PD: Paulina Daw
CG: Colin Greaves
TW: Thomas M Withers
JVvZ: Jet JCS Veldhuijzen van Zanten
AH: Alexander Harrison
HFrEF: Heart failure with reduced ejection fraction
HFpEF: Heart failure with preserved ejection fraction
HFmrEF: Heart failure with mid-range ejection fraction
CR: Cardiac rehabilitation
HF: Heart failure
