# Supplementary material for: A systematic review of provider-and system-level factors influencing the delivery of cardiac rehabilitation for heart failure
Source: BMC Health Serv Res. 2021 Nov 24;21:1267. doi: 10.1186/s12913-021-07174-w (PMC8611948; doi:10.1186/s12913-021-07174-w)
Supplement: Supplementary file 1 — Additional file 1. MEDLINE Ovid search strategy. [file 12913_2021_7174_MOESM1_ESM.docx]

Additional file 1. MEDLINE Ovid search strategy

1. exp heart failure/

2. heart failure.ab,ti.

3. HFrEF.ab,ti.

4. HFpEF.ab,ti.

5. HFmrEF.ab,ti.

6. 1 or 2 or 3 or 4 or 5

7. exp cardiac rehabilitation/

8. cardi* rehab*.ab,ti.

9. exp Exercise/

10. exercis*.ab,ti.

11. exp Rehabilitation/

12. rehab*.ab,ti.

13. enablement.ab,ti.

14. physical activit*.ab,ti.

15. 7 or 8 or 9 or 10 or 11 or 12 or 13 or 14

16. offer*.ab,ti.

17. exp Health Plan Implementation/

18. exp Implementation Science/

19. implement*.ab,ti.

20. exp "Referral and Consultation"/

21. refer*.ab,ti.

22. exp "Delivery of Health Care"/

23. exp "Delivery of Health Care, Integrated"/

24. deliver*.ab,ti.

25. exp Health Services Accessibility/

26. provi*.ab,ti.

27. 16 or 17 or 18 or 19 or 20 or 21 or 22 or 23 or 24 or 25 or 26

28. 15 and 27

29. barrier*.ab,ti.

30. exp "Attitude of Health Personnel"/

31. enabl*.ab,ti.

32. facilitat*.ab,ti.

33. factor*.ab,ti.

34. influenc*.ab,ti.

35. 29 or 30 or 31 or 32 or 33 or 34

36. 6 and 28 and 35

37. limit 36 to english language**

** The search terms were adapted as appropriate for the following databases Embase (OVID interface), PsycINFO (OVID interface), CINAHL Plus, and EThoS and ProQuest libraries
