# Supplementary material for: A systematic review of provider-and system-level factors influencing the delivery of cardiac rehabilitation for heart failure
Source: BMC Health Serv Res. 2021 Nov 24;21:1267. doi: 10.1186/s12913-021-07174-w (PMC8611948; doi:10.1186/s12913-021-07174-w)
Supplement: Supplementary file 2 — Additional file 2. Provider- and system-level barriers and enablers identified in the literature. [file 12913_2021_7174_MOESM2_ESM.docx]

Additional file 2. Provider- and system-level barriers and enablers identified in the literature

|  | Barriers and enablers identified in the literature |
| --- | --- |
| Non-patient barriers | Tradition of resting HF patients, lack of a mortality benefit with CR among HF patients, lack of evidence for alternatives to centre-based CR, a large variety of complex guidelines and position statements, guidelines providing no specific implementation details, CR not included in the local guidelines, poor healthcare professional education, organisational constraints (e.g. lack of facilities and time), high costs of CR, lack of equipment, high cost and shortage of multidisciplinary teams, exclusion from local commissioning agreements, lack of inclusion in the contract with the referring institutions, other people (e.g. HF specialist nurses) providing a similar service, lack of healthcare integration, governance split among several entities, nonalignment of payment incentives and/or information systems between organisations, lack of local patient pathways, lack of medical insurance cover, lack of integration of clinical algorithms into the Information and Communication Technology systems, professional isolation between departments, lack of minimal standards and consistency in HF management, lack of well-defined effective strategies for implementing CR, lack of referrals from healthcare professionals, lack of alternatives to centre-based CR, reduced number of operating centres, limited eligibility criteria, difficult to decide which programme patients should be referred to (where several programmes are available), extensive and confusing referral tests, professional's lack of knowledge, awareness, familiarity and attitude, ignorance among physicians with regard to the benefits of CR, overemphasis on procedural solutions, safety concerns, perception that HF patients are too ill for exercise, lack of support from healthcare professionals for HF rehabilitation, lack of knowledge on the benefits and safety of CR programmes, inefficient referral processes, poor flow of patient care from acute hospitals to outpatient and community follow-up, lack of strategies to improve healthcare professional’s condition-specific health literacy and referral processes, lack of flexibility in programme delivery. |
| Non-patient enablers | Data in favour of the beneficial effects and safety of CR in patients with HF, better tailoring of guidelines, inclusion of CR for the management of HF in contemporary clinical guidelines, combining and translating guidelines into clinical algorithms, development of cross-institutional guidelines, better implementation of the existing guidelines, education programmes for healthcare professionals, establishing inter-professional collaboration forums (e.g. working groups), creating possibilities for collective education (e.g. knowledge-sharing meetings), better strategies to improve physicians’ perceived benefits of CR, initiatives influencing awareness of the importance of CR (e.g. the Cardiac Rehabilitation Network of Ontario), improved insurance coverage or reimbursement, healthcare authorities to increase financial resources, refining multidisciplinary team responsibilities, providing integrated healthcare, collaboration with healthcare authorities, systematic inpatient referral, automatic referral by institutions, large-scale implementation of clinical algorithms, utilisation of the EXPERT tool (interactive decision-support), developing collaborative relationships between health professionals looking after HF patients, adding CR programmes to usual care programmes, incorporating CR into hospital performance measures, targeting non-referred populations, the rate of inpatient CR referral as a performance measure for the institution, choice between hospital-based rehabilitation and home-based individual programmes, innovative strategies and new delivery systems such as telemedicine, broadened eligibility, changing professionals’ attitudes regarding integration of care, encouragement of reluctant referrers, advertisement opportunities of rehabilitation programmes to healthcare professionals, flexibility within the rehabilitation programme delivery. |

CR, cardiac rehabilitation; HF, heart failure
